# Physiological Responses and Serum Metabolite Alterations in Grass Carp (*Ctenopharyngodon idellus*) Under Chronic Salinity Exposure

**DOI:** 10.3390/antiox14111287

**Published:** 2025-10-27

**Authors:** Xiajie Chen, Bing Li, Yiran Hou, Kepeng Wei, Linjun Zhou, Chengfeng Zhang, Liqiang Zhang, Jian Zhu, Rui Jia

**Affiliations:** 1 Wuxi Fisheries College, Nanjing Agricultural University, Wuxi 214081, China; chenxiajie@stu.njau.edu.cn (X.C.); lib@ffrc.cn (B.L.); houyr@ffrc.cn (Y.H.); 2 Key Laboratory of Integrated Rice-Fish Farming Ecology, Ministry of Agriculture and Rural Affairs, Freshwater Fisheries Research Center, Chinese Academy of Fishery Sciences, Wuxi 214081, China; lanqiuwangzi163@163.com (K.W.); zhoulinjun@ffrc.cn (L.Z.); zhangcf@ffrc.cn (C.Z.); zhangliqiang@ffrc.cn (L.Z.)

**Keywords:** salinity stress, metabolomics, hematology, oxidative stress, *Ctenopharyngodon idellus*

## Abstract

Salinity is a pivotal environmental factor that significantly influences the survival, growth, development, and reproduction of aquatic organisms. However, the characteristics of serum metabolites and their mechanistic roles in mediating the response of grass carp (*Ctenopharyngodon idellus*) to long-term salinity stress remain incompletely understood. Therefore, the present study exposed grass carp to different salinity levels (0, 4, and 8 g/L) for 60 days to evaluate the associated physiological alterations and metabolic responses. The results revealed that high salinity (8 g/L) significantly suppressed growth performance (*p* < 0.05), whereas low salinity (4 g/L) caused no significant reduction in growth or survival. Physiological analyses indicated that fish in the 8 g/L group exhibited markedly reduced levels of lactic acid and total protein, along with elevated concentrations of total cholesterol, triglycerides, glucose, and glutamic-oxaloacetic transaminase (*p* < 0.05). Serum ion homeostasis was also disrupted under high salinity, characterized by increased Ca^2+^, Na^+^, and Cl^−^ levels and decreased Mg^2+^ (*p* < 0.05). Furthermore, oxidative stress was evident in the high-salinity group through heightened activities of antioxidant enzymes (SOD, CAT, GPx), accumulation of oxidative damage markers (protein carbonyl, 8-OHdG) (*p* < 0.05). Metabolomic profiling identified 367 and 403 significantly altered metabolites in the 4 g/L and 8 g/L groups, respectively, primarily belonging to lipids and lipid-like molecules along with organic acids and derivatives. KEGG enrichment analysis revealed that these differential metabolites were chiefly involved in amino acid biosynthesis, glycerophospholipid metabolism, biosynthesis of unsaturated fatty acids, and glycine, serine, and threonine metabolism. Trend analysis further uncovered eight distinct expression patterns of metabolites across salinity gradients. These results provide novel insights into the metabolic adaptations of grass carp to salinity stress, demonstrating that high salinity induces oxidative stress, disrupts ion regulation, and drives extensive metabolic reprogramming. The study offers valuable theoretical support for improving salinity tolerance management in aquaculture and informs the selective breeding of salt-tolerant fish strains.

## 1. Introduction

Salinity serves as a critical physicochemical parameter in aquaculture systems, profoundly influencing fish physiology, growth performance, and overall health through its regulatory effects on osmoregulation, endocrine function, and immune competence. In freshwater aquaculture practices, salt (NaCl) has been extensively utilized for various purposes with notable beneficial effects. Its efficacy in disease control is well-documented, particularly for treating *ichthyophthiriosis* and preventing *saprolegniosis* in commercially important species like silver perch (*Bidyanus bidyanus*) [1]. The strategic use of salt as a transport additive has been shown to significantly alleviate physiological stress responses and improve survival rates [2], with studies reporting optimal concentrations such as 1 g/L for gold-spot catfish (*Ancistrus triradiatus*) transport [3]. Optimal salinity levels can enhance muscle quality in freshwater fish species, as demonstrated by the improved nutritional composition of snakehead (*Channa argus*) muscle tissue under appropriate salinity conditions (7.5 g/L) [4]. In addition, it has been reported that the isosmotic point of teleost fish is generally between 10–14 [5] or 9–12 g/L (as reviewed by PK Djiba et al.) [6]. The resting metabolic rate (RMR) of teleost is generally predicted to decrease as salinity increases toward their isosmotic point, due to reduced osmoregulatory. However, empirical studies reveal substantial interspecific variation, with responses ranging from RMR reduction to stable, linearly increasing, or bell-shaped patterns, depending on factors such as species, habitat, and specific salinity levels (as reviewed by PK Djiba et al.) [6].

Salinity also acts as an environmental stressor for fish, with varying tolerance levels among different freshwater species, making prolonged exposure to high-salinity conditions unsuitable for most freshwater fish. Early aquaculture studies indicated that the four major freshwater carps grew normally at salinities below 3 g/L. However, growth inhibition occurred in a species-specific manner at higher levels: silver carp (*Hypophthalmichthys molitrix*) and bighead carp (*Aristichthys nobilis*) were affected at 5 g/L, followed by grass carp (*Ctenopharyngodon idella*) at 8 g/L, and common carp (*Cyprinus carpio*) at 9 g/L [7]. For stinging catfish (*Heteropneustes fossilis*) fingerlings, salinity levels exceeding the optimal threshold of 6 g/L induced significant physiological stress, thereby increasing mortality and reducing growth rates [8]. Acute toxicity tests in juvenile silver catfish (*Rhamdia quelen*) showed complete mortality within 12 h at salinities ≥10 g/L, while survival remained stable at ≤4 g/L [9]. Unstable salinity also causes a reallocation of metabolic energy in fish. This is supported by an early study on common carp, which linked salinity above 6.5 g/L to reduced digestibility and increased nitrogenous excretion, thereby redirecting metabolic energy toward waste production and fecal loss [10]. Salinity (25 g/L) variation can lead to appetite suppression, reduced feed intake, and potentially lower feed conversion efficiency in the bester [11]. Salinity stress induces oxidative stress in fish and tissue damage [12,13]. Jiang et al. demonstrated that chronic exposure to saline condition (6 g/L) significantly compromised antioxidant enzyme activity, disrupted tissue morphology, and impaired osmoregulatory function in juvenile silver carp [14]. Notably, the physiological impacts of salinity stress follow a dose-dependent pattern. Moderate salinity variations within tolerable limits mainly cause energetic trade-offs, redirecting resources from growth to nitrogenous excretion, and other physiological maintenance processes [5]. In contrast, extreme salinity changes exceeding compensatory thresholds elicit severe pathological effects, such as inflammation, endoplasmic reticulum stress, and programmed cell death [15,16].

Blood plays a crucial role in reflecting the normal physiological state of animals. In fish, blood is closely linked to growth, reproduction, nutritional status, and disease. As a sensitive biosensor of organismal status, blood parameters undergo significant alterations in response to both physiological adaptations and pathological changes induced by environmental stressors. This characteristic establishes hematological analysis as a valuable tool for assessing nutritional status, environmental adaptability, and overall health condition in aquatic species [17]. Recent advances in serum/plasma metabolomics have significantly enhanced the capacity to monitor physiological responses through comprehensive characterization of small-molecule metabolites in blood serum. This high-throughput analytical approach facilitates the simultaneous detection of diverse metabolic intermediates, identification of novel biomarkers, and elucidation of mechanistic pathways involved in fish responses to environmental challenges. Early studies have utilized nuclear magnetic resonance (NMR)-based metabolomics to characterize the metabolic responses induced by environmental estrogens in plasma in rainbow trout (*Oncorhynchus mykiss*) [18]. Joanna Giebułtowicz et al. employed metabolomic profiling to characterize temporal variations in plasma metabolites associated with different sampling time points in common carp [19].

The grass carp is one of the most important freshwater aquaculture species [20]. Recent studies have demonstrated its limited tolerance to mildly saline–alkaline conditions, suggesting its potential as a viable candidate for brackish water aquaculture systems. This farming model not only expands potential aquaculture areas but also provides ecological benefits by improving saline–alkaline water systems. As a result, increasing research attention has focused on understanding the mechanisms of salinity adaptation and the physiological impacts of salt stress in grass carp. A 28-day culture trial demonstrated that the growth of grass carp was significantly inhibited at a salinity of 7.5 g/L [21]. When the salinity was raised to 8 g/L, decreased serum alpha-1 globulin and beta globulin levels were observed in grass carp [22]. Furthermore, exposure to 6 g/L salinity also induced significant physiological changes. Specifically, gill Na^+^-K^+^-ATPase activity showed a U-shaped response pattern [6], and the gut microbiota composition was altered, characterized by reduced probiotics and increased harmful bacteria [23]. It is noteworthy that existing evidence from studies on growth, osmoregulation, and physiological parameters suggests that the salinity in grass carp aquaculture should not exceed 8 g/L. However, the physiological responses and serum metabolic profiles of grass carp under prolonged salinity stress remain poorly understood. Therefore, this study investigates the effects of prolonged exposure to different salinity concentrations on physiological responses and serum metabolomics in grass carp. The findings will provide critical insights into the mechanisms of salinity adaptation, contribute to the development of salt-tolerant strains.

## 2. Materials and Methods

### 2.1. Animals, Experimental Design and Sampling

Healthy juvenile grass carp with an initial mean body weight of 100 ± 5 g were obtained from the Freshwater Fisheries Research Centre experimental farm (Wuxi, China). Prior to experimentation, fish were acclimated for two weeks in a recirculating aquaculture system under controlled laboratory conditions. Throughout the acclimation period, fish were fed twice daily with a commercial diet (Tongwei, Chengdu) at 2–3% of total biomass. The recirculating aquaculture system was operated using fully aerated freshwater as the water source. Measurements of temperature and dissolved oxygen (DO) were conducted with a YSI-DO 200 instrument (YSI Inc., Yellow Springs, OH, USA), while pH was quantified with an SG2 meter (METTLER TOLEDO, Greifensee, Switzerland). Water quality parameters were maintained as follows: temperature 28 ± 2 °C, pH 6.8–7.6, and dissolved oxygen >6 mg/L.

Following acclimation, the grass carp were randomly assigned to three salinity treatment groups: 0 g/L (control, SC), 4 g/L (S4), and 8 g/L (S8), with 60 days exposure. For each salinity level, three replicate tanks (12 fish per tank) were established. Throughout the experimental period, fish were fed the commercial diet following the previously described feeding protocol. Water quality maintenance included daily siphoning of residual feed and waste products, accompanied by a 20% water exchange. Following each water exchange, salinity levels were precisely restored through controlled addition of NaCl, with concentrations routinely verified using a calibrated handheld refractometer.

After the 60-day exposure period, four fish per tank were randomly sampled and anesthetized using 50 mg/L MS-222 (100 mg/L, Sigma, St. Louis, MO, USA) [24]. Blood samples were collected via caudal venipuncture. Serum was separated from blood via centrifugation (3500 r/min for 10 min at 4 °C). All samples were stored at −80 °C until analysis. All animal procedures conducted in this study were approved by the Animal Care Committee of the Freshwater Fisheries Research Center and strictly performed in compliance with relevant animal welfare guidelines.

### 2.2. Growth Performance

Specific growth rate (SGR), weight gain rate (WGR), survival rate (SR), and feed conversion ratio (FCR) of grass carp across salinity treatments were calculated based on measured body weight data, mortality records, and the number of remaining fish post cultivation. The formulae are defined as follows:

SGR = 100 × (LnW_2_ − LnW_1_)/T

WGR = 100 × (W_2_ − W_1_)/W_1_

SR = 100 × (final number/initial number)

FCR = total feed intake/total weight gain

where W_1_ and W_2_ represent initial body weight (g) and final body weight (g), and T denotes the experimental duration in days (d).

### 2.3. Serum Physiological Parameter Analysis

Serum was isolated from blood by centrifugation at 3000× *g* for 10 min at 4 °C, and all subsequent physiological assays were performed using commercial kits obtained from Nanjing Jiancheng Bioengineering Institute (Nanjing, China). Total protein (TP) was quantified using the bicinchoninic acid (BCA) method, with absorbance measured at 562 nm. Glucose (Glu) level was determined through an enzymatic reaction that yields a red-violet chromogen, detected at 505 nm. Lactate (LA) was analyzed via enzymatic generation of NADH, followed by a chromogenic reaction with specific reagents. Total cholesterol (TC) and triglycerides (TG) were measured using the COD-PAP method and GPO-PAP enzymatic assay, respectively, both with detection at 500 nm. Aspartate aminotransferase (GOT) activity was evaluated based on its catalytic role in the transamination between α-ketoglutarate and aspartate to form glutamate and oxaloacetate. Lactate dehydrogenase (LDH) activity was determined by measuring the absorbance at 450 nm of the brownish-red pyruvate dinitrophenylhydrazone hydrazone formed from the reaction of generated pyruvate with 2,4-dinitrophenylhydrazine.

Potassium ion (K^+^) concentration was determined by a turbidimetric method [25]. Chloride ion (Cl^−^) levels were assessed based on the formation of a chromogenic complex with mercuric thiocyanate, with absorbance measured at 436 nm. Magnesium (Mg^2+^) was quantified using a calmagite colorimetric method, measuring absorbance at 540 nm. Calcium (Ca^2+^) concentration was determined by reacting serum with methylthymol blue (MTB) in an alkaline medium to form a blue complex. Sodium ion (Na^+^) levels were analyzed turbidimetrically via reaction with potassium hexahydroxyantimonate in the presence of a dispersing agent, forming a uniform colloidal suspension. Na^+^, K^+^, and Cl^−^ assay kits were obtained from Nanjing Jiancheng Bioengineering Institute. Ca^2+^ and Mg^2+^ concentrations were measured with kits provided by Suzhou Geruisi Biotechnology Co., Ltd. (Suzhou, China). Cortisol levels were measured using commercial enzyme-linked immunosorbent assay (ELISA) kits from Shanghai Enzyme-linked Biotechnology Co., Ltd. (Shanghai, China), with absorbance read at 450 nm on a microplate reader.

### 2.4. Oxidative Stress Parameter Analyses

The activities of antioxidant enzymes and levels of oxidative stress markers were assessed using commercially available assay kits. Superoxide dismutase (SOD) activity was determined using the WST-1 method by measuring formazan formation at 450 nm. Total antioxidant capacity (T-AOC) was evaluated with the FRAP assay, based on the reduction of Fe^3+^ to Fe^2+^, and absorbance was read at 593 nm. Reduced glutathione (GSH) content was quantified by measuring the yellow chromogen derived from its reaction with 5,5′-dithiobis (2-nitrobenzoic acid) (DTNB). Glutathione peroxidase (GPx) activity was assayed using cumene hydroperoxide (Cum-OOH) as substrate and DTNB as chromogen. Catalase (CAT) activity was determined based on the catalytic decomposition of hydrogen peroxide (H_2_O_2_) to water and oxygen, accompanied by the formation of a red chromogen. Protein carbonyl content was determined by measuring absorbance at 370 nm following derivatization with 2,4-dinitrophenylhydrazine (DNPH). Malondialdehyde (MDA) levels were measured via the thiobarbituric acid reactive substances (TBARS) method at 532 nm. The content of 8-hydroxy-2′-deoxyguanosine (8-OHdG) was analyzed using an enzyme-linked immunosorbent assay (ELISA). SOD, T-AOC, GSH, and protein carbonyl assay kits were obtained from Nanjing Jiancheng Bioengineering Institute. GPx activity was measured using assay kits from Suzhou Geruisi Biotechnology Co., Ltd. Kits for MDA and CAT were sourced from Beyotime Biotechnology (Nantong, China). ELISA kits for 8-OHdG were supplied by Shanghai Enzyme-linked Biotechnology Co., Ltd.

### 2.5. Serum Metabolomics Analysis

For metabolite extraction, 100 μL serum were mixed with 400 μL of methanol/acetonitrile solution (1:1, *v*/*v*). The mixtures were incubated at −20 °C for 30 min, followed by centrifugation at 14,000× *g* and 4 °C for 20 min. The supernatant was carefully transferred to clean tubes and lyophilized using a vacuum concentrator. Prior to LC-MS analysis, the dried extracts were reconstituted in 100 μL of acetonitrile/water (1:1, *v*/*v*), vortex-mixed for 30 s, and centrifuged at 14,000× *g* for 15 min at 4 °C. The final supernatant was then prepped for instrumental analysis [26].

The acquired mass spectrometry data were processed using ProteoWizard software (version 3.0.6428) for format conversion to .mzML files. Subsequent data analysis was performed with the online XCMS platform (v3.7.1) for peak alignment, retention time correction, and peak area extraction. To ensure comprehensive metabolite detection, both positive and negative modes were employed during analysis. Initial data exploration was conducted through principal component analysis to visualize inter-group variations. For more refined group discrimination, we implemented OPLS-DA, a multivariate statistical approach that maximizes class separation. The model’s validity was confirmed through cross-validation and permutation tests. Metabolites showing significant alterations were selected based on dual criteria: variable importance in projection (VIP) scores exceeding 1.0 and statistically significant (*p* < 0.05) from Student’s *t*-tests. These differentially expressed metabolites were subsequently annotated against the KEGG pathway database to elucidate affected biological pathways.

### 2.6. Statistical Analysis

Data were analyzed using SPSS Statistics 26.0 (SPSS Inc., Chicago, IL, USA) and GraphPad Prism 9.5 (GraphPad Software, San Diego, CA, USA). Normality and homogeneity of variances were assessed via Shapiro–Wilk and Levene tests, respectively. Differences in growth performance and physiological parameters across salinity groups were evaluated by one-way analysis of variance (ANOVA) followed by LSD post hoc tests, with statistical significance defined at *p* < 0.05.

## 3. Results

### 3.1. Growth Performance

After 60 days of salinity exposure, the 8 g/L group exhibited significantly lower FBW, WGR, and SGR compared to both the 0 g/L and 4 g/L groups, while demonstrating a significantly higher FCR (*p* < 0.05; Table 1). While the 4 g/L group showed marginal decreases in growth parameters relative to controls, these variations did not reach statistical significance. Notably, the 8 g/L salinity group experienced 2.78% mortality, while both the 0 g/L and 4 g/L groups maintained 100% survival throughout the experimental period.

### 3.2. Physiological Parameters

**Figure 1:**
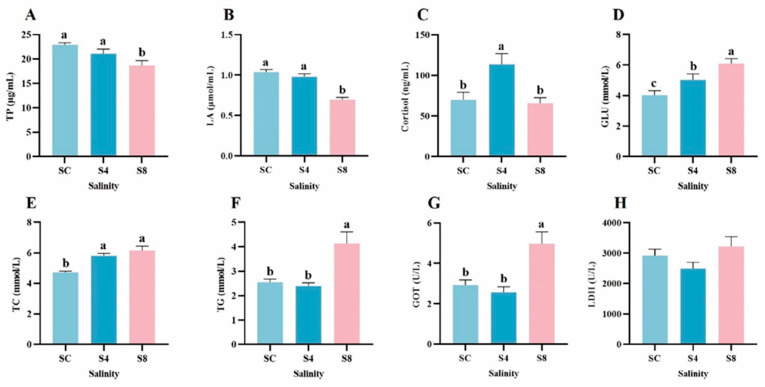
Changes in serum physiological parameters of grass carp after 60-day salinity exposure. (**A**) TP, (**B**) LA, (**C**) Cortisol, (**D**) GLU, (**E**) TC, (**F**) TG, (**G**) GOT, (**H**) LDH. Data are presented as means ± SEM (*n* = 12). Different superscript letters above bars denote significant differences (*p* < 0.05) among groups for each parameter. SC, 0 g/L salinity; S4, 4 g/L salinity; S8, 8 g/L salinity.

Following 60-day salinity exposure, significant physiological alterations were observed in serum of grass carp (Figure 1). The 8 g/L group demonstrated significantly reduced LA and TP concentrations compared to 0 g/L group (*p* < 0.05; Figure 1A,B). Conversely, this group exhibited marked elevations in levels of TC, TG, and GOT (*p* < 0.05; Figure 1E–G). GLU content rose significantly with escalating salinity (*p* < 0.05; Figure 1G). Cortisol levels exhibited a biphasic response to salinity, peaking significantly in the 4 g/L group (*p* < 0.05) before returning to normal levels in the 8 g/L treatment group (Figure 1C). Additionally, LDH activity remained unaffected by salinity exposure, showing no significant differences among groups (*p* > 0.05; Figure 1H).

### 3.3. Serum Ion Content

**Figure 2:**
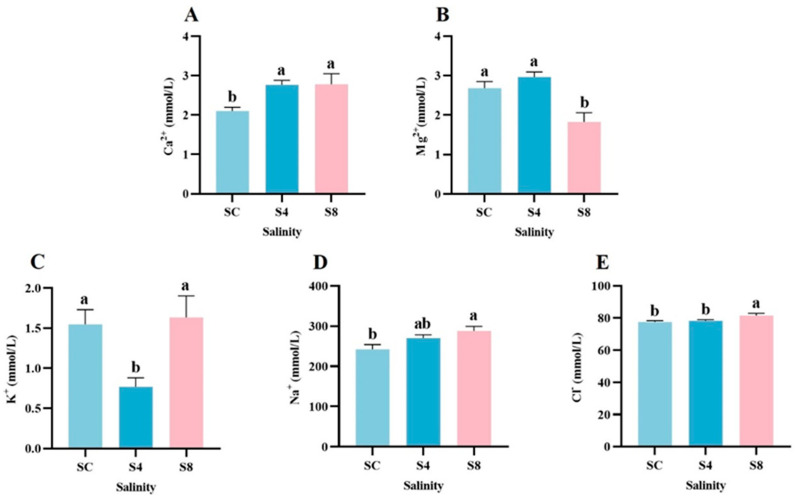
Changes in serum ion levels of grass carp after 60-day salinity exposure. (**A**) Ca^2+^, (**B**) Mg^2+^, (**C**) Na^+^, (**D**) K^+^, (**E**) Cl^−^. Data are presented as means ± SEM (*n* = 12). Different superscript letters above bars indicate significant differences (*p* < 0.05) among groups for each parameter. SC, 0 g/L salinity; S4, 4 g/L salinity; S8, 8 g/L salinity.

Serum ion profiles showed significant salinity-dependent alterations (Figure 2). Compared to the 0 g/L group, the 8 g/L group showed significantly decreased Mg^2+^ levels along with increased Ca^2+^, Na^+^, and Cl^−^ concentrations (*p* < 0.05; Figure 2A–C,E). In contrast, K^+^ levels in the 4 g/L group were significantly lower than in both the 0 g/L and 8 g/L groups (*p* < 0.05; Figure 2D).

### 3.4. Oxidative Stress Parameters

**Figure 3:**
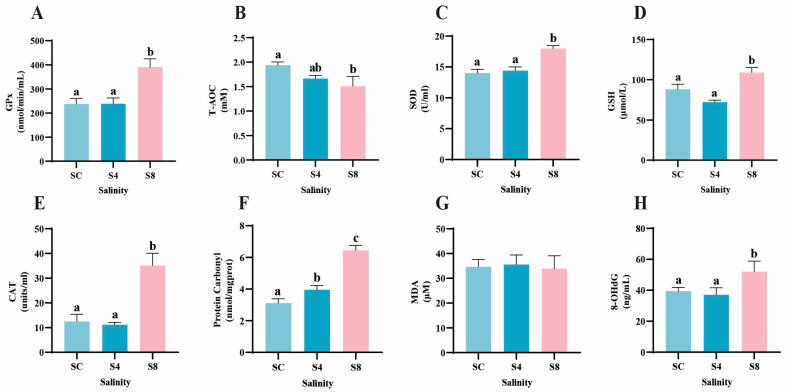
Changes in serum antioxidant status of grass carp after 60-day salinity exposure. (**A**) Glutathione peroxidase (GPx), (**B**) Total antioxidant capacity (T-AOC), (**C**) Superoxide dismutase (SOD), (**D**) Reduced glutathione (GSH), (**E**) Catalase (CAT), (**F**) Protein carbonyl, (**G**) Malondialdehyde (MDA), (**H**) 8-hydroxy-2′-deoxyguanosine (8-OHdG). Data are presented as means ± SEM (*n* = 12). Different superscript letters above bars denote significant differences (*p* < 0.05) among groups for each parameter. SC, 0 g/L salinity; S4, 4 g/L salinity; S8, 8 g/L salinity.

After 60-day salinity exposure, the 8 g/L group showed significant increases in serum oxidative stress markers compared to 0 g/L group. SOD, CAT, GPx activities, and GSH levels were all significantly elevated, along with oxidative damage markers protein carbonyl and 8- 8-OHdG (*p* < 0.05; Figure 3A,C–F,H). In contrast, T-AOC was significantly reduced in the 8 g/L group (Figure 3B). In the 4 g/L exposure group, only protein carbonyl levels were significantly elevated (*p* < 0.05), while other measured parameters remained unchanged. Additionally, no significant differences in MDA levels were observed among different groups (*p* > 0.05; Figure 3G).

### 3.5. Serum Metabolomics

#### 3.5.1. Serum Metabolites Composition

**Figure 4:**
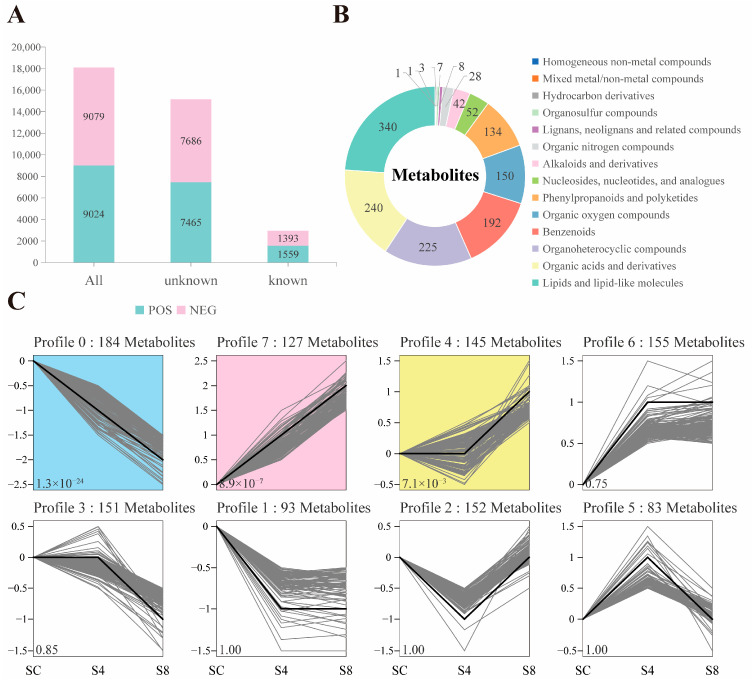
Serum metabolic profile of grass carp. (**A**) Metabolite identification results in positive and negative ionization modes; (**B**) Quantitative distribution and categorical classification of all annotated metabolites after ion mode merging; (**C**) Trend analysis of identified metabolites across sequential salinity groups (SC, S4, S8). Colored profiles denote significantly enriched trends (*p* < 0.05). SC, 0 g/L salinity; S4, 4 g/L salinity; S8, 8 g/L salinity.

In grass carp serum, 18,103 metabolites were detected across positive and negative ionization modes, with 15,151 unidentified and 2952 metabolites structurally characterized (Figure 4A). Identified metabolites predominantly comprised lipids and lipid-like molecules (340), organic acids and derivatives (240), organoheterocyclic compounds (225), benzenoids (192), organic oxygen compounds (150), and phenylpropanoids and polyketides (134) (Figure 4B).

Trend analysis was performed to cluster metabolite expression patterns across longitudinal sample sets (≥3 time points) based on their expression profiles. From the structurally characterized metabolites, 1862 were initially filtered, resulting in 1090 metabolites that exhibited eight distinct trend profiles across the sequential groups (SC vs. S4 vs. S8). Among these, Profile 0, Profile 4, and Profile 7 were identified as significantly enriched trend modes (*p* < 0.05; Figure 4C).

Specifically, with increasing salinity, 427 metabolites showed an upward trend (Profiles 4, 6, and 7), while 428 metabolites exhibited a downward trend (Profiles 0, 1, and 3). Additionally, 152 metabolites demonstrated a pattern of initial decrease followed by increase, and 83 metabolites displayed an initial increase followed by decrease (Figure 4C).

#### 3.5.2. Differential Metabolites Analysis

**Figure 5:**
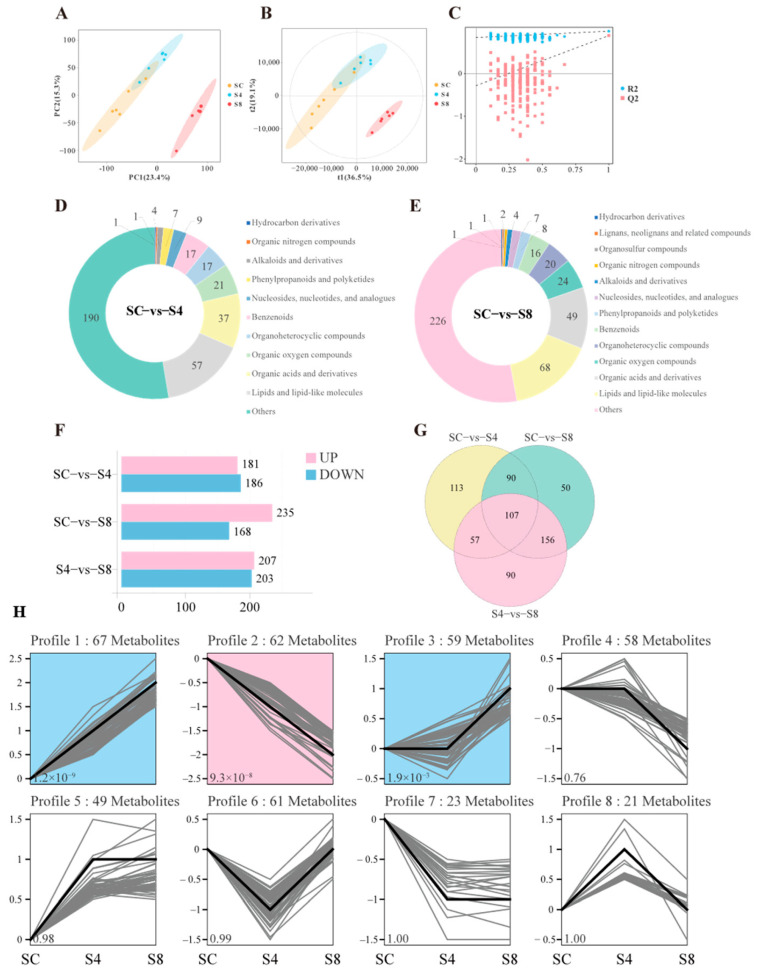
Serum metabolomic analysis of grass carp following salinity exposure. (**A**) PCA score plot of metabolites. (**B**) PLS-DA score plot of metabolites. (**C**) Permutation test for validating the PLS-DA model. (**D**,**E**) Categorical classification of differential metabolites between SC vs. S4 and SC vs. S8, respectively. (**F**) Differential metabolites identified across comparison groups. (**G**) Venn diagram illustrating shared and unique differential metabolites among SC, S4, and S8 groups. (**H**) Trend analysis of differential metabolites across sequential salinity groups (SC, S4, S8). Colored profiles represent significantly enriched trend modes (*p* < 0.05). SC, 0 g/L salinity; S4, 4 g/L salinity; S8, 8 g/L salinity.

Following the integration of data from both positive and negative ionization modes, principal component analysis (PCA) showed clear separation between the 0 g/L group and the 8 g/L treatment group (Figure 5A). Partial least squares-discriminant analysis (PLS-DA) further indicated distinct metabolite segregation between these two groups (Figure 5B), with the reliability of the model validated by permutation tests (Figure 5C). In contrast, no significant separation was observed between the 0 g/L and 4 g/L groups, suggesting that a salinity level of 8 g/L has a more substantial influence on the serum metabolic profile of grass carp.

After 60 days of salinity exposure, significant alterations were observed in the serum metabolome of grass carp. Compared to the 0 g/L group, the 4 g/L salinity group showed 181 upregulated and 186 downregulated metabolites, while the 8 g/L group exhibited 235 upregulated and 168 downregulated metabolites (Figure 5F). Differential metabolites between the 0 g/L and 4 g/L groups (Figure 5D) were primarily classified into the following categories: lipids and lipid-like molecules (57), organic acids and derivatives (37), organooxygen compounds (21), organoheterocyclic compounds (17), and benzenoids (17). Between the 0 g/L and 8 g/L groups (Figure 5E), the major categories included lipids and lipid-like molecules (68), organic acids and derivatives (49), organooxygen compounds (24), organoheterocyclic compounds (20), and benzenoids (16).

Venn diagram analysis illustrated the shared and unique differential metabolites across the SC-vs.-S4, SC-vs.-S8, and S4-vs.-S8 comparisons (Figure 5G). A total of 107 differential metabolites were common to all groups, while 113 were unique to SC vs. S4, 50 to SC vs. S8, and 90 to S4 vs. S8. These metabolites displayed eight distinct trend profiles across the sequential salinity groups. Among these, Profile 1, Profile 2, and Profile 3 were identified as significantly enriched trend modes (*p* < 0.05; Figure 5H). Specifically, with increasing salinity, 148 significantly differential metabolites showed an upward trend (Profiles 2, 4, and 7), whereas 175 exhibited a downward trend (Profiles 1, 3, and 5). Additionally, 61 metabolites demonstrated an initial decrease followed by an increase, and 21 metabolites displayed an initial increase followed by a decrease (Figure 5H).

#### 3.5.3. Metabolic Pathway Analysis

**Figure 6:**
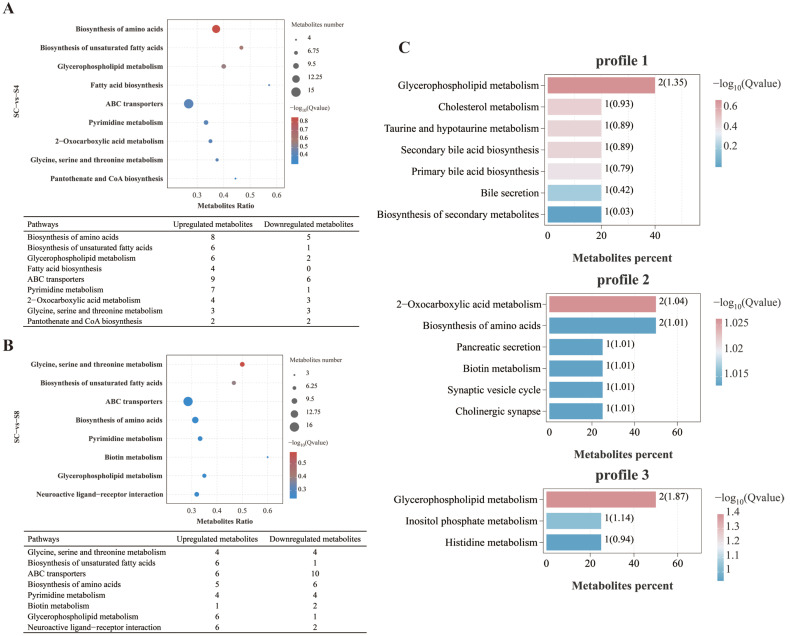
Serum metabolomic analysis of grass carp among SC, S4 and S8 groups after salinity exposure. (**A**) Top enriched KEGG pathways of differential metabolites: SC vs. S4. (**B**) Top enriched KEGG pathways of differential metabolites: SC vs. S8. (**C**) Primary KEGG pathways enriched by metabolites from significantly enriched trend modes. SC, 0 g/L salinity; S4, 4 g/L salinity; S8, 8 g/L salinity.

Enrichment analysis revealed significant pathway-level alterations in metabolite profiles under salinity stress. Compared to the 0 g/L group, the 4 g/L group showed marked enrichment of differential metabolites in biosynthesis of amino acids (*p* = 0.001), biosynthesis of unsaturated fatty acids (*p* = 0.003), and glycerophospholipid metabolism (*p* = 0.006) (Figure 6A). In the 8 g/L group, significant enrichment was observed in glycine, serine and threonine metabolism (*p* = 0.002), biosynthesis of unsaturated fatty acids (*p* = 0.006), and ABC transporters (*p* = 0.016) (Figure 6B). Furthermore, KEGG enrichment analysis of metabolites associated with significantly enriched trend modes highlighted predominant involvement in glycerophospholipid metabolism and 2-oxocarboxylic acid metabolism (Figure 6C).

**Figure 7:**
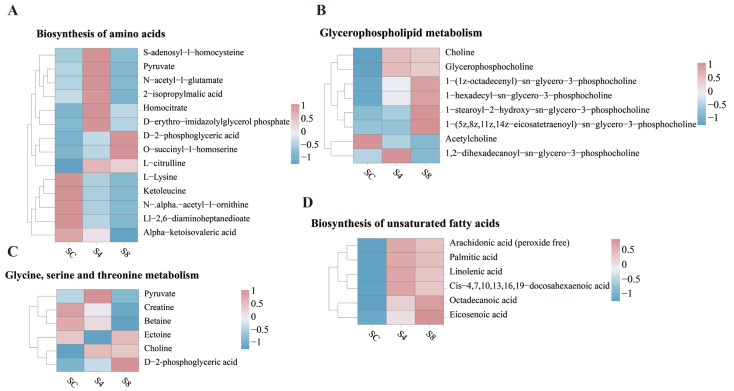
Alterations in relative abundance of metabolites within key signaling pathways. (**A**) Amino acid biosynthesis pathway. (**B**) Glycerophospholipid metabolism pathway. (**C**) Glycine, serine and threonine metabolism pathway. (**D**) Unsaturated fatty acid biosynthesis pathway.

Among the top ten enriched pathways, four were consistently altered under both 4 g/L and 8 g/L salinity. In amino acid biosynthesis, S-adenosyl-L-homocysteine, pyruvate, *N*-acetyl-L-glutamate, and 2-isopropylmalic acid were significantly increased in the 4 g/L group compared to the 0 g/L group, but their relative abundance decreased under 8 g/L salinity. The relative abundance of L-lysine, ketoleucine, *N*-α-acetyl-L-ornithine, LL-2,6-diaminoheptanedioate, and α-ketoisovaleric acid was consistently downregulated with increasing salinity. D-2-Phosphoglyceric acid and O-succinyl-L-homoserine exhibited a sustained upregulation trend (Figure 7A).

In glycine, serine, and threonine metabolism, betaine and choline were consistently downregulated, while D-2-phosphoglyceric acid was upregulated. Compared to the 0 g/L group, pyruvate was significantly elevated in the 4 g/L group, whereas ectoine was markedly downregulated at this salinity level (Figure 7B).

In the glycerophospholipid metabolism pathway, 1,2-dihexadecanoyl-sn-glycero-3-phosphocholine showed an initial increase followed by a decrease across salinity groups. Conversely, 1-(1Z-octadecenyl)-sn-glycero-3-phosphocholine and 1-hexadecyl-sn-glycero-3-phosphocholine exhibited sustained upregulation with increasing salinity, and acetylcholine was consistently downregulated. 1-Stearoyl-2-hydroxy-sn-glycero-3-phosphocholine and 1-(5Z,8Z,11Z,14Z-eicosatetraenoyl)-sn-glycero-3-phosphocholine were significantly upregulated in the 8 g/L group. Choline and glycerophosphocholine were more markedly upregulated in the 4 g/L group (Figure 7C).

Similarly, in the unsaturated fatty acid metabolism pathway, all detected metabolites showed upregulated trends. Among these, arachidonic acid (peroxide free), palmitic acid, linolenic acid, and cis-4,7,10,13,16,19-docosahexaenoic acid were more significantly upregulated in the 4 g/L group, while eicosenoic acid and octadecanoic acid exhibited more pronounced upregulation in the 8 g/L group (Figure 7D).

#### 3.5.4. Signature Differential Metabolites

Following screening of metabolites exhibiting significant changes, we identified 10 metabolites that were upregulated and 10 that were downregulated in response to increasing salinity. These metabolites may serve as potential biomarkers in the blood of grass carp for assessing salinity adaptation.

**Figure 8:**
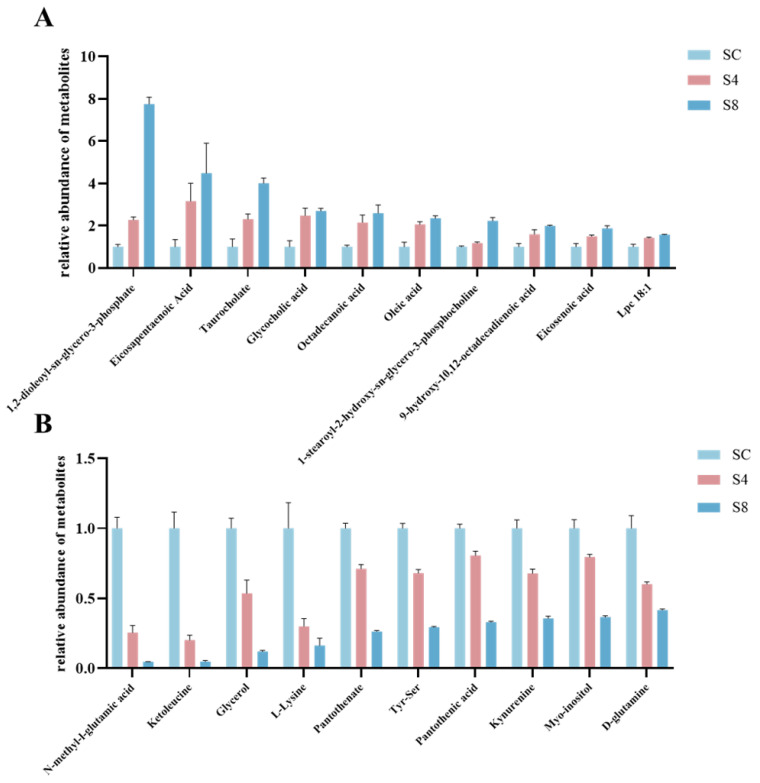
Serum metabolomic analysis of grass carp among NC, 4 g/L, and 8 g/L groups after salinity exposure. (**A**) Consistently increasing metabolite trends in 4 g/L and 8 g/L groups versus NC. (**B**) Consistently decreasing metabolite trends in 4 g/L and 8 g/L groups versus NC. The data were normalized prior to analysis.

Specific metabolites exhibited consistent linear increasing or decreasing trends across both 4 g/L and 8 g/L salinity groups (Figure 8A,B). Among these, 1,2-dioleoyl-sn-glycero-3-phosphate showed the most pronounced upregulation in the 8 g/L group, while *N*-methyl-L-glutamic acid was the most downregulated. Metabolites with increasing abundance were predominantly classified as lipids and lipid-like molecules. In contrast, those with decreasing trends were primarily organic acids and derivatives, as well as organic oxygen compounds (Table 2).

## 4. Discussion

Salinity adaptation in fish is a complex physiological process that encompasses multiple systems, including growth, osmoregulation, ion regulation, metabolism, immune response and endocrine function. Freshwater fish maintain a hyperosmotic state relative to their ambient environment, exhibiting high sensitivity to salinity variations. Salinity fluctuations directly challenge this inherent ionic and physiological homeostasis, inducing metabolic dysfunction and a series of pathological consequences—notably tissue damage, immune impairment, and exacerbated oxidative stress [17,27,28]. Under abrupt environmental changes, the regulation of energy and material metabolism becomes critical for acclimation, as it modulates the supply of metabolic energy and key biosynthetic intermediates [29]. Blood parameters serve as important indicators of physiological stress and overall health in fish exposed to nutritional or environmental challenges [30,31]. However, most existing studies on fish salinity adaptation have not comprehensively integrated aspects of energy metabolism, stress responses, and endocrine regulation. Moreover, targeted metabolomic analyses of blood components remain limited. In this context, the present study aims to clarify the effects of salinity stress through systematic serum metabolomic profiling

### 4.1. Salinity Stress Inhibited Growth Performance

It has been confirmed that some freshwater fish can survive at elevated salinities; however, growth performance is often adversely affected beyond species-specific thresholds [32]. For example, common carp reared at 5 g/L maintain normal growth, whereas growth rates are suppressed at 15 g/L [33]. Salinity stress has been shown to disrupt energy balance in fish, requiring greater energy expenditure to maintain physiological homeostasis (as reviewed by Marcos Tavares-Dias) [17]. The lowered growth performance is also correlated with reduced feed intake and feed efficiency ratio under high-salinity conditions [34]. Rahmah et al. further demonstrated that in hybrid red tilapia, elevated salinity reduces feed intake and utilization efficiency, thereby compromising digestive and metabolic functions [35]. In this study, compared to the 0 g/L group, the 4 g/L group showed a non-significant decline in FBW, FCR, SGR, and WGR. In contrast, the 8 g/L group exhibited significant reductions in FBW, SGR, and WGR, along with an increase in FCR. These results indicate that chronic exposure to high salinity has substantial detrimental effects on the growth of grass carp.

The reduced survival observed in the 8 g/L group in our study is consistent with reports of decreased survival in common carp at 10 g/L [36]. Unstable environmental conditions, including high salinity, can impair physiological status by inducing metabolic dysfunction and oxidative stress, as reported by Sun et al. and Zhang et al. [12,37].

### 4.2. The Effect of Salinity Stress on Physiological Parameters

Variations in aquatic salinity directly modulate hematological parameters in fish, making them pivotal biomarkers for assessing physiological status. These parameters are influenced by a range of intrinsic and extrinsic factors, including seasonal fluctuations, species-specific traits, size and ontogenetic stage, genetic background, stocking density, geographical distribution, and habitat characteristics. As a result, hematological indices serve as essential biomarkers in physiological, pathological, and toxicological assessments, providing critical insights into fish health, nutritional status, and environmental adaptability [38]. Research in fish hematology is significant not only for advancing fundamental knowledge in piscine physiology, but also for its practical applications in aquaculture management and disease prevention [25].

Serum glucose serves as a key physiological indicator that reflects energy metabolism and stress status in fish. Under salinity stress, fish often elevate blood glucose levels by enhancing glycogenolysis and gluconeogenesis to meet the additional energy demands [39]. Similarly, serum cortisol—a major glucocorticoid hormone and a primary biomarker of the stress response in fish—is released following activation of the hypothalamic–pituitary–interrenal (HPI) axis in response to stressors such as salinity change. Elevated cortisol levels typically indicate acute stress and stimulate the mobilization of energy reserves via gluconeogenesis, glycogenolysis, and lipolysis [40]. Furthermore, cortisol serves as a critical osmoregulatory hormone that upregulates branchial Na^+^/K^+^/2Cl^−^ cotransporter (NKCC) and Na^+^/K^+^-ATPase (NKA) to promote ion secretion and uptake [41]. The rise in plasma glucose observed in stenohaline carp (*Cyprinus carpio*) transferred from FW to brackish water reflects a metabolic shift that diverts energy from growth toward stress response processes [42]. Comparable responses have been reported in red sea bream (*Pagrus major*), where low-salinity stress led to significant increases in plasma cortisol, glucose, total cholesterol, and triglycerides [43]. In the present study, we found a significant increase in serum GLU levels in grass carp at a salinity of 8 g/L. This result is consistent with observations in common carp at 9 g/L salinity, suggesting that elevated glucose levels may contribute to the maintenance of plasma osmolality within a constant range [44]. Furthermore, the physiological stress response and increased nitrogenous excretion induced by high salinity exposure may impose an additional energy demand, driving the rise in circulating glucose levels.

Interestingly, cortisol levels were elevated at 4 g/L salinity but remained unchanged compared to the control group at 8 g/L in the present study. The increase at 4 g/L implies chronic mild stress, likely induced by suboptimal osmotic conditions that lead to persistent HPI axis activation. This finding is partially consistent with the results of Kammerer et al. [45], who observed that tilapia showed a rapid increase in cortisol levels after being transferred to a seawater environment, followed by a gradual decline over several days under sustained salinity challenge. The elevated cortisol level may function to aid in the regulation of ion balance for freshwater fish facing salinity stress [46,47]. Additionally, cortisol can regulate ion transport by modulating the activity of gill Na^+^/K^+^-ATPase [48]. Taken together, findings from previous studies and our present work demonstrate that differential stress responses to salinity are influenced by both the concentration and duration of exposure, which significantly modulate hormone secretion patterns and physiological adaptation. We hypothesize that after 60 days of exposure, fish in the 8 g/L group may have undergone partial physiological adaptation, entering a state of chronic stress homeostasis. In this state, cortisol levels may return to near baseline, while other physiological costs—such as sustained high energy expenditure—remain elevated. However, the precise molecular and endocrine mechanisms underlying this response require further investigation.

Previous studies have reported a significant reduction in serum total protein in goldfish (*Carassius auratus*) under increasing salinity [49]. Our data are consistent with this trend, demonstrating a decrease in TP in grass carp following prolonged salinity stress. This reduction may be attributed to the enhanced utilization of proteins for energy production in response to the adverse stimuli. Protein synthesis is energy-consuming process. The chronic salinity stress disrupts the innate homeostatic balance of grass carp, likely triggering a reprioritization of energy expenditure. In this state, energy may be diverted away from costly anabolic processes like protein synthesis, leading to the observed reduction in serum total protein. Concurrently, our findings revealed a significant decrease in serum lactate level after 60 days of exposure to 8 g/L salinity. In stellate sturgeon (*Acipenser stellatus Pallas*) under combined salinity and ammonia stress, decreased lactate levels were associated with higher ammonia concentrations, suggesting reduced anaerobic metabolic activity—a phenomenon potentially related to hemoglobin function [50]. This decline may reflect an adaptive strategy wherein chronically stressed fish reduce anaerobic activity (e.g., intense locomotion) to conserve energy and limit stress accumulation.

In the present study, a significant increase in GOT activity was observed in fish exposed to 8 g/L salinity. Similar results were also observed in *Carassius auratus* after 4–12 g/L salinity exposure [51]. As GOT is an intracellular enzyme, its elevated in plasma is commonly used as a biomarker for liver injury, often resulting from toxicant-induced hepatic tissue damage and subsequent enzyme leakage [52]. For instance, elevated GOT levels have been reported in juvenile olive flounder (*Paralichthys olivaceus*) [53], Nile tilapia (*Oreochromis niloticus*) [54], and gibel carp (*Carassius gibelio*) [55] following exposure to toxicants. We propose that long-term salinity stress causes functional and/or structural damage to the liver of grass carp, increasing hepatocyte membrane permeability and facilitating the release of GOT into the circulation. Moreover, the heightened demand for energy under salinity stress enhances protein catabolism and hepatic gluconeogenesis. Since GOT is a key enzyme in these metabolic pathways, its increased synthesis and turnover may further contribute to the elevated serum levels, in addition to its leakage due to hepatocellular damage [56].

### 4.3. The Effect of Salinity Stress on Ion Content

The adaptive capacity of fish to varying environmental salinities fundamentally depends on their ability to regulate ion uptake and excretion, as well as to maintain hydro-mineral homeostasis [57]. Under elevated salinity conditions (6–12 g/L), common carp increased blood Na^+^, Cl^−^, and K^+^ levels to maintain hydromineral balance, suggesting both a common response to salinity challenge and a species-specific tolerance limit of approximately 9–12 g/L [58]. Similarly, in Mozambique tilapia (*Oreochromis mossambicus*), salinity stress led to significant increases in serum osmolality and concentrations of Na^+^, K^+^, and Cl^−^, reflecting an adaptive response that supports fluid balance and ion transport homeostasis [59]. Consistent with these findings, our study demonstrated an elevation in serum Na^+^ (an increase of 20.83%) and Cl^−^ (an increase of 8%) concentrations in grass carp under high-salinity exposure. This response aligns with the general ionoregulatory adaptation pattern observed in many aquatic species under non-optimal salinity conditions, wherein active ion uptake helps maintain osmotic equilibrium [60]. Our results are consistent with a recent acute study in grass carp, which also observed a significant elevation in serum Na^+^ and Cl^−^ concentrations at 7 g/L salinity [61]. We speculate that although 8 g/L salinity is near the isosmotic point, it nonetheless acts as a stressor for freshwater-adapted grass carp, thereby disrupting their inherent homeostatic balance and leading to ion dysregulation. Interestingly, while a significant decrease in K^+^ concentration was observed at 4 g/L salinity, its restoration to near-baseline levels at 8 g/L suggests that grass carp may activate progressively effective compensatory mechanisms with prolonged exposure, potentially involving enhanced ion-regulatory function in gill and renal tissues.

In this study, serum Ca^2+^ levels increased significantly under long-term salinity stress (4 and 8 g/L). This finding is consistent with a previous report on Nile tilapia, which also observed elevated blood Ca^2+^ at higher salinities of 10 and 15 g/L [62]. The rise in Ca^2+^ levels may originate from impaired calcium homeostasis, primarily caused by dysfunctional calcium transport in the gill and gut [63]. In contrast, serum Mg^2+^ concentration exhibited a clear decreasing trend in the present study. This finding is consistent with observations in Mozambique tilapia, where serum Mg^2+^ levels were significantly lower in fish acclimated to 25% and 50% seawater compared to those in freshwater [64]. As an essential cofactor for key enzymes such as alkaline and acid phosphatases and hexokinase [65], the decline in Mg^2+^ may reflect adaptive modifications in energy metabolism and enzymatic reactions under sustained salinity stress. Although short-term studies have reported transient elevation of Mg^2+^ [66], its persistent decrease observed here suggests that long-term salinity stress may elicit ion regulatory strategies distinct from acute responses. The depletion of Mg^2+^ may indicate altered activity or demand of certain enzyme systems during prolonged acclimation.

### 4.4. Salinity Stress-Induced Oxidative Stress

Salinity stress in fish has been closely linked to the enhanced generation of reactive oxygen species (ROS), resulting in significant oxidative damage [67]. To counteract ROS-mediated damage, fish rely on their endogenous antioxidant defense system, which neutralizes free radicals and enhances overall stress resilience [68]. Central to this protective response are core scavenging enzymes—including SOD, CAT, and GPx—which constitute the first line of defense against oxidative stress. Under salinity stress, SOD activity in *Penaeus monodon* rose by approximately 50%, suggesting an adaptive response to superoxide accumulation [69]. Similarly, in European sea bass (*Dicentrarchus labrax*), elevated blood SOD under low-salinity stress was proposed to prevent oxidation of adrenaline, a hormone that increases during stress [70]. Juvenile silver pomfret (*Pampus argenteus*) subjected to low salinity showed synergistic upregulation of SOD and CAT activities in the kidney and muscle [71]. Beyond salinity stress, GPx activity increased significantly in common carp following metal exposure, highlighting its role in scavenging lipid peroxides [72]. Elevated GSH levels have been frequently reported in Amazonian brycon (*Brycon amazonicus)* under HgCl_2_ exposure [73], often associated with enhanced substrate uptake and enzyme biosynthesis to synergistically alleviate oxidative stress. In the present study, the upregulation of SOD, CAT, GPx, and GSH levels in serum indicated the presence of oxidative stress under chronic salinity exposure. Changes in salinity can trigger a range of physiological responses in aquatic animals, such as increased levels of stress-related hormones, accelerated energy metabolism, and disruption of ion regulation. These alterations are closely linked to the generation of ROS [74]. The observed increases in these biomarkers reflect an activation of the antioxidant system, representing an adaptive response that helps counteract salinity-induced oxidative stress.

8-OHdG, a product of oxidative DNA damage, is widely recognized as a reliable biomarker for cellular oxidative stress [75]. Its formation is closely associated with ROS generation, and elevated tissue levels of 8-OHdG reflect intensified oxidative stress [76]. Long-term fluoride exposure significantly increased 8-OHdG levels in the kidneys and livers of rats, demonstrating fluoride-induced DNA damage [75]. Similarly, cadmium exposure in rainbow trout led to elevated 8-OHdG across multiple tissues, indicating systemic DNA oxidative damage [77]. Protein carbonyl content serves as another key indicator of oxidative damage, representing irreversible structural modification and functional loss in proteins due to oxidation [78]. Excessive ROS production has been shown to cause protein oxidation in tissues of rainbow trout [79]. In zebrafish (*Danio rerio*) exposed to ibuprofen and aluminum, significantly elevated protein carbonyl levels indicated pronounced protein oxidative damage [80,81]. Likewise, nitrite-exposed grass carp exhibited increased protein carbonylation in gill tissues, likely resulting from ROS overproduction [82]. Consistent with these findings, grass carp under long-term salinity stress exhibited significant increases in both serum 8-OHdG and protein carbonyl content, indicating substantial oxidative damage. This suggests that salinity stress induces excessive ROS formation, overwhelming the antioxidant defense system and leading to oxidative attack on macromolecules.

### 4.5. The Effect of Salinity Stress on Metabolic Functions

Amino acids play central roles in critical biological processes, including nitrogen metabolism, protein synthesis, energy production, and immune regulation [83]. Environmental stressors are known to disrupt amino acid metabolism in aquatic species. For instance, carbonate alkalinity stress markedly altered amino acid metabolism in Dabry’s loach (*Paramisgurnus dabryanus*) [84]; and high ammonia stress impaired amino acid transport and metabolism in juvenile yellow catfish (*Pelteobagrus fulvidraco*) [85]. Metabolomic studies further highlight the adaptive reorganization of amino acid metabolism under stress. Ammonia stress in the ornate rock lobster (*Panulirus ornatus*) led to significant changes in levels of L-isoleucine, valine, proline, *N*-acetylglycine, and glycine [86]. Similarly, in Pacific white shrimp (*Litopenaeus vannamei*), heat stress was found to predominantly affect phenylalanine metabolism and the biosynthesis of aromatic amino acids [87]. These findings indicate that aquatic organisms modulate distinct amino acid pathways in response to different stressors to maintain physiological homeostasis. In this study, salinity stress significantly influenced amino acid metabolism in grass carp, with pronounced enrichment in pathways such as glycine, serine, and threonine metabolism and amino acid biosynthesis. These amino acids contribute critically to osmoregulation, energy supply, and antioxidant defense [88,89,90]. Beyond serving as protein precursors, amino acids act as substrates for ATP generation—fueling ion transporters like Na^+^/K^+^-ATPase [91,92]. Furthermore, they contribute significantly to maintaining redox homeostasis, immune modulation, and signal transduction [93]. Therefore, the marked enrichment of amino acid metabolism under salinity stress reflects a strategic reallocation of amino acid resources to enhance cytoprotection, sustain energy metabolism, and mitigate oxidative damage during salinity adaptation.

Lipids are essential nutrients that play critical roles in organismal growth, structural integrity, and physiological function, in addition to serving as a major source of energy [86]. Under environmental stress, aquatic organisms often enhance their adaptability by modulating fatty acid composition to maintain cellular membrane fluidity [94,95,96]. As the primary constituents of cell membranes, glycerophospholipids exhibit concentration changes that reflect adjustments in membrane composition and permeability, thereby directly influencing cellular physiology [86]. Numerous studies have indicated that saline–alkaline stress markedly disrupts lipid metabolism in aquatic species [97,98]. For instance, Song et al. observed that Nile tilapia under high saline–alkaline conditions rely increasingly on lipid metabolism to meet energy demands [99]. Similarly, Ding et al. demonstrated that prolonged exposure to carbonate alkalinity stress in crucian carp aggravates renal cell membrane damage and disrupts glycerophospholipid and sphingolipid metabolism [39]. Notably, unsaturated fatty acids (UFAs) play an essential role in maintaining and regulating cellular functions. Their accumulation helps restore membrane fluidity, sustain membrane-associated enzyme activity, and enhance membrane tension to improve cellular stress tolerance [100]. Studies on kuruma shrimp (*Marsupenaeus japonicus*) under cold stress revealed upregulation of UFA biosynthesis, indicating their dual role in membrane stabilization and energy metabolism [95]. Consistent with these findings, our study demonstrates significant enrichment of lipid metabolism pathways—particularly glycerophospholipid and unsaturated fatty acid metabolism—in grass carp under salinity stress. Multiple studies have indicated that salinity challenge appears to activate UFA and glycerophospholipid biosynthesis, which are critical for stress adaptation [101,102]. These results further support the notion that lipid metabolic reprogramming is a key adaptive strategy in fish. Specifically, grass carp may adjust glycerophospholipid profiles to stabilize membrane structures and accumulate UFAs to alleviate oxidative damage, maintain membrane functionality.

### 4.6. The Effect of Salinity Stress on Key Metabolites

Following 60 days of salinity exposure, significant alterations were observed in the serum metabolome of grass carp, with lipid metabolites exhibiting particularly pronounced changes. For instance, oleic acid and eicosapentaenoic acid (EPA) showed a linear increase in response to elevated salinity levels (4 and 8 g/L). It is well-established that salinity stress modulates membrane properties such as permeability, fluidity, structural integrity, and the activity of membrane-embedded transporters [103]. In this context, oleic acid contributes to cellular ion homeostasis and enhances membrane fluidity [104], while EPA is closely associated with salinity adaptation and stress resistance—elevated EPA levels improve membrane fluidity and modify the lipid microenvironment [105]. Our results demonstrate a persistent upregulation of various lipids and lipid-like molecules in the serum, indicating a sustained metabolic reprogramming that supports long-term acclimation. The observed response may be driven by two primary mechanisms: (1) Membrane remodeling: The increased synthesis of specific phospholipids and unsaturated fatty acids—such as oleic acid and EPA—is essential for preserving membrane fluidity and structural integrity. This reorganization is critical for maintaining the function of ion transporters and channels involved in osmoregulation. (2) Energy provision: Lipid catabolism likely serves as an important energy source, supporting the heightened metabolic demands of ion homeostasis under salinity stress.

In this study, a sustained downregulation trend was also observed in various organic acids and derivatives, as well as organooxygen compounds, in grass carp under long-term salinity stress. Particularly noteworthy was the significant reduction in myo-inositol, a compound with crucial physiological regulatory functions. Myo-inositol has been extensively demonstrated to enhance salinity tolerance, immune competence, growth performance, and stress resistance in aquatic species [106]. As a key compatible osmolyte, myo-inositol normally accumulates under osmotic imbalance to alleviate cellular damage induced by hyper- or hypoosmotic conditions and participates in modulating osmotic stress signaling pathways [107]. Studies have shown that knockdown of genes involved in myo-inositol biosynthesis and the consequent decrease in myo-inositol concentration significantly impair osmoregulatory capacity in species such as turbot (*Scophthalmus maximus*) [108]. We postulate that the decrease in myo-inositol occurs as environmental salinity nears the isosmotic point, leading to a reduced osmotic differential and a consequent downregulation in the demand for organic osmolytes like myo-inositol. The specific mechanisms responsible for this reduction, however, require further elucidation.

Furthermore, L-lysine, an essential amino acid for both mammals and fish, serves not only as a limiting amino acid in protein biosynthesis but also as a precursor for L-carnitine synthesis, thereby indirectly participating in the β-oxidation of fatty acids and playing a vital role in lipid metabolism and energy supply [109]. Previous studies have indicated that various environmental stressors can influence lysine metabolism. For instance, in Pacific white shrimp, combined ammonia and heat stress resulted in decreased L-lysine levels concurrent with disruptions in lipid metabolic pathways [87]. Similarly, Mozambique tilapia showed reduced L-lysine content under salinity stress, further supporting the involvement of this amino acid in adaptive responses to environmental challenges [110]. The sustained downregulation of L-lysine observed in the present study may reflect an energy-saving strategy, in which the organism reduces the synthesis and accumulation of certain non-essential metabolites under prolonged salinity exposure. On the other hand, it may also indicate impaired lipid metabolism, specifically through compromised fatty acid transport and β-oxidation via the lysine-derived carnitine pathway.

### 4.7. Integrative Physiology and Metabolomics Informing the Precision Breeding of Salt-Tolerant Grass Carp

This study elucidates the adaptive mechanisms of grass carp to chronic salinity stress by profiling serum physiology and metabolomics and identifies critically altered metabolites. This work lays a foundation for the selective breeding of salt-tolerant grass carp germplasm. (1) The key serum metabolites and pathways identified in this study serve as predictive biomarkers for salt tolerance. Their levels can be quantified in juveniles as a prognostic tool, enabling early selection and dramatically shortening the breeding period for salt-tolerant strains. (2) The integration of physiological and metabolomic data facilitates the development of a composite salt tolerance score. This integrated score can effectively stratify individuals based on their salt tolerance capacity. (3) Metabolomic analysis has elucidated the core metabolic pathways involved in the grass carp’s response to salinity stress. This knowledge informs the development of targeted breeding strategies, such as Marker-Assisted Selection (MAS) or gene editing, for selecting parent fish with superior genotypes in these critical pathways.

## 5. Conclusions

This study integrated hematological indices and metabolomic profiling to elucidate the mechanisms underlying the response of grass carp to prolonged salinity stress. The results demonstrate that increasing salinity levels significantly disrupt growth performance, physiological homeostasis, and serum metabolic patterns. Following 60 days of exposure, salinity at 8 g/L markedly inhibited growth, manifesting as suppressed weight gain, SGR, and reduced survival. As a significant environmental stressor, salinity induced oxidative stress and imposed substantial osmoregulatory costs, resulting in elevated energy allocation and consequent growth restriction. Metabolomic analyses revealed extensive reorganization of the serum metabolome under chronic high-salinity conditions, most prominently in amino acid and lipid metabolic pathways. Specifically, lipids and lipid-like molecules were persistently upregulated, whereas organooxygen compounds, organic acids, and derivatives were downregulated. These metabolic reprogramming phenomena indicate that grass carp modulates lipid mobilization and amino acid metabolism to counteract osmotic imbalance and oxidative damage; however, such adaptations may concurrently provoke cellular structural impairment and dysregulation of energy metabolism, collectively exacerbating the observed reduction in growth and survival under high salinity. These findings provide novel mechanistic insights into the metabolic basis of salinity adaptation in grass carp and contribute a theoretical foundation for developing salt-tolerant varieties and improving aquaculture management strategies in salinity-affected environments.

## Figures and Tables

**Table 1:** Effect of salinity stress on the growth performance of grass carp after 60 days of exposure.

Parameters	SC	S4	S8
IBW (g)	103.08 ± 0.70	105.17 ± 1.71	102.31 ± 0.91
FBW (g)	261.55 ± 18.06 ^a^	248.34 ± 15.65 ^a^	133.73 ± 9.91 ^b^
SGR (%/d)	1.51 ± 0.11 ^a^	1.39 ± 0.11 ^a^	0.49 ± 0.11 ^b^
WGR (%)	139.92 ± 11.80 ^a^	145.40 ± 12.11 ^a^	30.76 ± 5.48 ^b^
SR (%)	100	100	97.22
FCR	1.54 ± 0.25 ^b^	1.70 ± 0.36 ^b^	5.81 ± 1.90 ^a^

The results are expressed as the mean ± SEM. For each row, means with different letters as superscripts indicate a statistical significance among different groups (*p* < 0.05). SC, 0 g/L salinity; S4, 4 g/L salinity; S8, 8 g/L salinity.

**Table 2:** Biological categories of metabolites with consistently increasing or decreasing trends.

Tendency	Metabolites	Metabolite Class
Upregulation	1,2-dioleoyl-sn-glycero-3-phosphate	Lipids and lipid-like molecules
	Eicosapentaenoic Acid	Lipids and lipid-like molecules
	Taurocholate	Lipids and lipid-like molecules
	Glycocholic acid	Lipids and lipid-like molecules
	Octadecanoic acid	Lipids and lipid-like molecules
	Oleic acid	Lipids and lipid-like molecules
	1-stearoyl-2-hydroxy-sn-glycero-3-phosphocholine	Lipids and lipid-like molecules
	9-hydroxy-10,12-octadecadienoic acid	Lipids and lipid-like molecules
	Eicosenoic acid	Lipids and lipid-like molecules
	Lpc 18:1	Lipids and lipid-like molecules
Downregulation	*N*-methyl-l-glutamic acid	Organic acids and derivatives
	Ketoleucine	Organic acids and derivatives
	L-Lysine	Organic acids and derivatives
	Pantothenate	Organic acids and derivatives
	D-glutamine	Organic acids and derivatives
	Tyr-Ser	Organic acids and derivatives
	Myo-inositol	Organic oxygen compounds
	Glycerol	Organic oxygen compounds
	Kynurenine	Organic oxygen compounds
	Pantothenic acid	Organic oxygen compounds

## Data Availability

All data are contained within the main manuscript.
